# Let us lead: community leadership in the AIDS response is its fundamental pillar for success

**DOI:** 10.1002/jia2.26196

**Published:** 2023-11-30

**Authors:** Florence Riako Anam, Sbongile Nkosi, Meirinda Sebayang, Maximina Jokonya, Keren Dunaway, Tariq El Alaoui

**Affiliations:** ^1^ GNP+ Kisumu Kenya; ^2^ GNP+ Johannesburg South Africa; ^3^ Jaringan Indonesia Positif (JIP) Jakarta Indonesia; ^4^ Y+ Global Cape Town South Africa; ^5^ ICW Global Buenos Aires Argentina; ^6^ MENA Community Morocco Morocco

1

The global movement of people living with and affected by HIV has evolved into a first‐of‐a‐kind demonstration of community leadership that connects the grassroots to the global community, powerfully shaping the agenda of the AIDS response. We are known for our powerful presence and voice, actively mobilizing and influencing global policy, financing and action. Over time, we have built infrastructure to support even the “hard to reach” communities with what they need to live high‐quality, healthy lives [1, 2].

Our movement enshrines the Denver Principles developed 40 years ago, asserting the rights and self‐determination of people living with HIV. Likewise, the Greater Involvement of People with HIV (GIPA) principle promotes the meaningful participation of people living with HIV in decision‐making, not as service recipients or patients, but as equal partners in contributing to the global AIDS response goals. We strongly believe in “nothing for us without us [3].”

Communities set the standard for meaningful engagement and hold partners accountable to them. Our organizations make deliberate investments in young people's growth through Her Voice Fund, the GNP+ and Y+ Global Young and Emerging Leaders programme (YEL), and other similar activities, to ensure that our sustained advocacy passes down through generations, firmly grounded in our history and fundamental principles [4, 5].

Our work and expertise support our informed engagement in other global concerns, directly and indirectly influencing the world's ability to address pressing issues, including COVID‐19 [6], climate change, universal health coverage, pandemic preparedness prevention and response, and global public investment.

The 2021 UN Political Declaration on HIV/AIDS affirms the critical role we play in achieving the 30‐80‐60 targets, which state that by 2025, community‐led organizations should deliver 30% of testing and treatment services, 80% of HIV prevention services for populations at high risk of infection and 60% of programmes to support societal enablers [7].

Globally, networks founded by people living with HIV and key populations have evolved into national, regional and global civil society organizations, building and nurturing institutionalized leadership. Our work has been possible because policy makers, researchers and governments at the country to global levels have progressively expanded opportunities for community engagement, participation and leadership. Community‐focused investment by key donors who provide core funding for community‐led organizations, organizational strengthening and building of capacity, and technical expertise enable networks from national to global levels to exist as reputable institutions. We have grown in our technical capacity to engage in policy and programme work as equal partners. This investment in community‐led networks has grown the quality of our advocacy, engagement and community leadership [8].

We have and continue to do this through the representation of people living with and affected by HIV both nationally and globally in the governance of UNAIDS, the Global Fund and other key global partners like UNITAID, the International AIDS Society and donors like PEPFAR, ensuring informed policy and implementation.

An example is the Jaringan Indonesia Positif (JIP), which has been actively involved in shaping HIV prevention policies at both national and regional levels. Through its participation in the Global Fund Country Coordinating Mechanism, JIP led on planning for the implementation of community programmes like the COVID‐19 Response Mechanism (C19RM). A further powerful example is the experience of the International Community of Women Living with HIV (ICW), whose advocacy on forced and coerced sterilization, alongside the Kenya Legal and Ethical Issues Network on HIV & AIDS (KELIN), contributed to legal victory on these issues in Kenya [9]. Meanwhile, the community‐led People Living with HIV Stigma Index is a trusted source for information and detailed data on how stigma and discrimination affect the lives of people living with HIV and cause varied barriers to accessing HIV treatment services, resulting in preventable deaths. The Stigma Index is being used by UNAIDS and governments to address stigma‐related barriers to HIV prevention and treatment services.

People living with and affected by HIV lead national community‐led monitoring (CLM) initiatives. CLM is an important accountability mechanism that uses the power of evidence and perspectives gathered from communities and countries to advocate for improvements in laws and policies, attitudes and practices. Community‐led service delivery continues to ease the burden of health facilities while increasing access to services, particularly for the most marginalized. This is done through health awareness and treatment literacy, demand creation, task sharing of services for HIV and sexually transmitted infections (STI) testing, and facilitation of differentiated service delivery to improve uptake and retention of prevention services like pre‐exposure prophylaxis (PrEP) and HIV treatment [1].

Community movement‐building and activism continue to be the foundation of our power. We embrace our diversity and come together to demand attention and prioritization for things that matter to us. From ACT UP in the United States to Treatment Action Campaign in South Africa, community‐led campaigns create change. The Undetectable = Untransmittable (U = U) campaign is motivating treatment access and adherence worldwide, and the GNP+ led “Not a Criminal” campaign brings together networks of people living with HIV and key populations to demand evidence‐based legislation to protect our communities from stigma, discrimination, criminalization and violence.

However, despite the clear evidence of the benefits of community leadership, our ability to lead is significantly hampered by shifting global health governance and donor priorities, an unprecedented global political and social polarization demonstrated by a rapidly shrinking civic space, and regression in laws and policies affecting human rights. Global movements and organizations of and for people living with HIV and key populations often work with very little to no support and in an environment that continuously questions our capacity and credibility. Until recently, our involvement in the national and global AIDS response has been very tokenistic, our role has remained largely voluntary and our significant contributions have not been counted or documented [10].

*We are leaders*. We are rooted in our belief that people living with and affected by HIV are experts in our own lives. We are bold and effective leaders of the AIDS response in our communities and at national and global levels. We are guided by a rights‐based approach, promoting self‐determination and full participation in decisions that affect our lives [2]. We have unmatched experience, expertise and reach. LET US LEAD.

## COMPETING INTERESTS

The authors declare no competing interests.

## AUTHORS’ CONTRIBUTIONS

FRA and SN drafted the Viewpoint. MS, MJ, KD and TEA reviewed the Viewpoint. All authors approved the contents of this Viewpoint.

## DISCLAIMER

The contents in this Viewpoint represent the views of the authors and members and do not necessarily reflect the views of the authors’ affiliated organizations.

## References

[1] UNAIDS . Community‐led AIDS responses. Geneva: UNAIDS; 2018. Available from: https://www.unaids.org/sites/default/files/media_asset/community‐led‐aids‐responses_en.pdf

[2] GNP+ strategic plan 2023–2026: for our health, human rights, and dignity. Amsterdam: Global Network of People Living with HIV; 2021. Available from: https://gnpplus.net/resource/gnp‐strategic‐plan‐2023‐2026‐for‐our‐health‐human‐rights/

[3] The Denver Principles Project . The Denver principles. 1983. Available from: https://www.unaids.org/sites/default/files/media/documents/1983_denver‐principles_en.pdf

[4] UNAIDS . Denver principles 40 years on. 2023. Available from: https://www.unaids.org/en/resources/presscentre/featurestories/2023/june/20230626_denver‐principles‐40‐years‐on#:~:text=Forty%20years%20on%20from%20this,though%20we%20are%20passive%20agents

[5] Global Health Governance Programme . Background. Available from: https://governance‐principles.org/background/

[6] Y+ Global . We Matter: value us—ethical engagement guideline. Available from: https://www.yplusglobal.org/resources/we‐matter‐value‐us‐ethical‐engagement‐guideline

[7] GNP+ . Beyond LIVING: COVID‐19. Amsterdam: Global Network of People Living with HIV; 2020. Available from: https://gnpplus.net/wp‐content/uploads/2020/07/BeyondLIVING_COVID‐19_English.pdf

[8] United Nations General Assembly . Political declaration on HIV and AIDS: ending inequalities and getting on track to end AIDS by 2030. New York: United Nations; 2021. Available from: https://www.unaids.org/en/resources/documents/2021/2021_political‐declaration‐on‐hiv‐and‐aids

[9] KELIN . Court victory: landmark ruling exposes discrimination in forced sterilization of HIV‐positive women. Nairobi: KELIN; 2021. Available from: https://www.kelinkenya.org/court‐victory‐landmark‐ruling‐exposes‐discrimination‐in‐forced‐sterilization‐of‐hiv‐positive‐women/

[10] Doctors without borders : Community‐led interventions: urgent and sound investment for an effective HIV response in West and Central Africa MSF; 2020. Available from: https://www.doctorswithoutborders.org/latest/msf-support-needed-community-led-hiv-programs-west-and-central-africa

